# Lower mitochondrial DNA copy number in ATTRV50M Swedish carriers

**DOI:** 10.1186/s13023-026-04645-3

**Published:** 2026-10-06

**Authors:** Gabriella Johannson, Malin Olsson, Marina Rubio Garcia, Anders Olofsson, Intissar Anan

**Affiliations:** 1 Department of Public Health and Clinical Medicine, Umeå University, Umeå, Sweden; 2 Department of Medical Biosciences, Umeå University, Umeå, Sweden; 3 Department of Microbiology, Umeå University, Umeå, Sweden

**Keywords:** Amyloidosis, ATTR, Transthyretin, Hereditary, Mitochondrial DNA copy number, V30M, V50M, qPCR, Oxidative stress, Sweden

## Abstract

**Background:**

Hereditary transthyretin (TTR) amyloidosis caused by TTR p.Val50Met (ATTRV50M) is a progressive systemic disease caused by misfolding of the protein transthyretin (TTR). The conversion of TTR into an amyloidogenic state has recently been found to be strongly influenced by a non-native disulfide bond, thus also a redox environment. Variation in mitochondrial DNA (mtDNA) copy number serves as an indirect marker of redox imbalance and mitochondrial dysfunction. MtDNA is moreover strictly maternally inherited, supporting previous findings where maternal inheritance of the pathogenic variant also results in both a more severe disease and an earlier onset. Considering these aspects, we aimed to investigate mtDNA levels in Swedish TTRp.Val50Met carriers.

**Methods:**

304 individuals were divided into three groups: symptomatic and asymptomatic TTR p.Val50Met carriers, and healthy non-carrier controls. Relative mtDNA copy number was quantified from buffy coat samples using multiplex qPCR.

**Results:**

Symptomatic TTR p.Val50Met carriers showed a significantly lower copy number (*p* = 0.002) compared to the asymptomatic carriers. When stratified by sex, a significantly lower mtDNA copy number in symptomatic compared to asymptomatic carriers was observed only among males (*p* = 0.028). Furthermore, a significant difference in copy number (< 0.001) could be demonstrated in ≥ 50 year-olds (considered late onset in Swedish ATTRV50M population) betweensymptomatic and asymptomatic TTR p.Val50Met carriers, which could not be seen in < 50 year-olds (Swedish early onset).

**Conclusion:**

These findings indicate an association between lower mtDNA copy numbers and symptomatic disease in Swedish TTR p.Val50Met carriers, suggesting potential mitochondrial involvement in disease progression.

## Background

Transthyretin amyloidosis (ATTR) is a systemic disease caused by a pathological aggregation of the protein transthyretin (TTR). ATTR amyloidosis comes in two forms: non-hereditary (ATTRwt) and hereditary (ATTRv). ATTRv amyloidosis is more frequently diagnosed than ATTRwt and it is the most common type of hereditary amyloidoses, affecting 1 in 100 000 people world-wide. Because ATTRv amyloidosis is a systemic disease, the symptoms are diverse and range from a general autonomic and/or peripheral neuropathy to more organ-specific cardiopathy, kidney-, and liver dysfunction [1]. Progressive symmetrical length-dependent peripheral neuropathy is usually the debut symptom and can often be seen years before any other symptoms emerge. Other common manifestations are carpal tunnel syndrome, gastrointestinal dysfunction, renal failure, glaucoma, and perhaps the more acutely severe and life-threatening symptom, cardiopathy, causing arrythmias with subsequent cardiac failure [2–4].

In ATTRv amyloidosis, TTR has a single-point pathogenic variant, bringing instability to the tetrameric TTR structure and ameliorating the amyloidotic nature of the protein. There are more than 200 pathogenic TTR variants associated with ATTR amyloidosis currently reported, and the number continues to expand as additional variants are identified and deposited in public databases. The most common variant is a substitution from valine to methionine at position 50 of the mature protein, p.Val50Met (ATTRV50M, historically called ATTRV30M) [5]. The way through which TTR becomes able to form fibrils is, in a simplified way, by disassociation of the tetramer, which undergoes a conformational change that in the end allows the subunits to bind to each other in a structured way, forming fibrils [6]. The TTR sequence has a cysteine that recently has been found to be important regarding its conversion into an amyloidogenic precursor state in vivo [7]. Recent research has shown that the variant TTR molecules, such as the V50M variant are particularly flexible. This flexibility can, under the right environmental circumstances, facilitate a conformational change which shortens the distances between the cysteines of two subunits, allowing them to form a disulfide bond [8]. Incidentally, this creates locked forms of highly amyloidogenic structures [9, 10]. The formation of a disulfide bond requires an oxidising environment [8] which allows for speculation of a potential disturbance of the redox balance that encourages TTRV50M formation in carriers of TTR p.V50M that eventually develop ATTRV50M amyloidosis. Building on this, it is important to consider the role of mitochondria, both as key regulators of the cellular redox environment and as unique carriers of maternally inherited DNA. The redox balance is therefore a central factor in ATTRV50M pathogenesis, and mitochondria play a principal role as they are both a major source and regulator of reactive oxygen and nitrogen species (ROS/RNS). Variation in mitochondrial DNA (mtDNA) copy number often reflects mitochondrial function and the degree of oxidative stress. Thus, it could be hypothesised that alterations in mtDNA levels may serve as an indirect marker of disturbances in the cellular redox environment, which in turn could influence TTR stability and its tendency to misfold.

To expound, mtDNA is unique in being strictly maternally inherited. This is of particular interest in ATTRV50M amyloidosis, since several studies have suggested that disease penetrance and age of onset may differ depending on whether the pathogenic variant is inherited from the mother or the father [11, 12]. Together, these aspects make mtDNA copy number an especially relevant parameter to study, as it may connect mitochondrial function, redox status, and inheritance patterns to the variability of disease expression among carriers.

The redox environment is a paramount factor in many cellular reactions and necessary for normal cellular function. It is regulated by a complex network of internal systems as well as outside sources. The mitochondrial respiration is one of the internal systems that has a large role in this balance, as it is a producer of large amounts of reactive oxygen and nitrogen species (ROS and RNS).

Mitochondria are vital organelles most notably responsible for energy production in mammalian cells in the form of adenosine triphosphate (ATP). Contrary to all other organelles, mitochondria are in possession of their own distinct genomic material, so-called mitochondrial DNA (mtDNA) and are almost exclusively inherited from the mother [13]. Compared to nuclear DNA, which is diploid, the separate mtDNA is polyploid, and there can be an up or down regulation of the mtDNA leading to multiple copies depending on the tissue or the organism’s energy needs. As stem cells differentiate, the mtDNA copy number is high as a consequence of increased mtDNA replication and will lower as the cells differentiate and later mature [14]. In mature cells, the amount of mtDNA copies is tissue specific, and an overall stable maintenance of the (tissue specific) correct level of mtDNA is associated with healthy cell aging [15, 16]. However, an abnormally up or down regulated copy number is also indicative of mitochondrial dysfunction and oxidative stress [17, 18]. In many pathological conditions, a trend of abnormal mitochondrial function has been encountered. Indeed, mitochondrial dysfunction, and the connected mtDNA copy number variation, is associated with a wide range of pathological conditions, such as various forms of cancer, familial diseases, as well as general aging and mortality [21, 22]. Lowered levels of mtDNA have been correlated with aging, as well as unfavourable outcomes of cardiovascular disease and endometrial cancer [19]. Mortality following stroke has been linked to both a lower and a higher copy number [20]. Additionally, in many neurodegenerative diseases such as Alzheimer’s disease (AD), Parkinson’s disease (PD), and multiple sclerosis (MS), a lower number of mtDNA copies has been noted, suggesting mitochondrial dysfunction could be involved in the pathogenesis [23–26]. A study done on a Portuguese population of TTR p.V50M carriers, showed that they have a higher mean copy number of mtDNA compared to controls, especially in carriers with early age of onset (< 40 years old) [27]. These associations have deemed mtDNA copy number a proxy for mitochondrial function and is currently being investigated as a potentially interesting future biomarker for many conditions.

Even though the amyloidosis affects people all over the world, there are clustering areas with a higher prevalence of ATTRv. ATTRV50M amyloidosis is associated with areas in Japan, Brazil, Portugal, and Sweden. The inheritance pattern is autosomal dominant but, conflictingly, there is a variation of disease penetrance and age of onset. This variation can be seen both between different variants of ATTRv amyloidosis and between different clustering areas considering even the same pathogenic variant [28]. In Portugal, Japan and Brazil, there is a high penetrance, and the mean age of onset is 20–40 years of age, while Sweden, as well as some sporadic cases in Japan, has a lower penetrance and a higher mean age of onset at 50 years of age [1, 3, 29, 28].

Previous studies show that development of ATTRV50M amyloidosis can be associated with a lower age of onset and usually with a more severe degree of symptoms than the affected parent experienced. This association was most pronounced when the affected parent was a mother who had an affected son [11, 12]. Findings like these, as well as the fact that variation in the mtDNA copy number occurs in many other pathological conditions, leads to the speculation of a possible mitochondrial involvement in ATTRv amyloidosis as well.

## Methods

### Aim

Based on prior findings about oxidative environment expediting amyloid formation, the effect on anticipation depending on maternal inheritance, as well as abnormal mtDNA levels being a marker for various pathologies and dysfunctional redox environment, we aim to investigate mtDNA copy number variation in the Swedish TTR p.V50M carrier population. Specifically, we aim to elucidate whether there are differences in mtDNA copy number in symptomatic- and asymptomatic TTR p.V50M carriers compared to a non-carrier control group.

### Study population and DNA purification

Blood samples from a total of 304 individuals from northern Sweden, with ages ranging from 21.3 to 92.4 (mean age = 59.6, standard deviation = 14.4) years were included in this study. Age of participant implies age at blood sampling. The study population was selected so that it would be as age and sex matched as possible, while still having a large enough sample size. See Table 1 for a detailed description of the study population and genotype groups.

Prior to this study, all blood samples were centrifuged (16 min,1600 rcf at room temperature) to isolate their buffy coats, then stored at -80 °C until DNA purification. The DNA to be used in the quantification of mtDNA copy number was purified from buffy coat using FlexiGene DNA kit (250) (Qiagen), following the kit- provided protocol. The purified DNA samples were incubated for 3–4 days on a rocker at room temperature (done to fully dissolve the DNA in the purification kit-supplied resuspension buffer). Finally, the samples’ concentrations were determined using NanoDrop spectrophotometer ND-1000 (SaveenWerner).

The individuals were divided into three categories: symptomatic TTR p.V50M carriers (*n* = 165), asymptomatic TTR p.V50M carriers (*n* = 69), and non-carrier controls (*n* = 70). The symptomatic group consisted of individuals fulfilling clinical diagnostic criteria for ATTRv amyloidosis based on the presence of the TTR p.Val50Met variant, compatible clinical manifestations, and biopsy-confirmed amyloid deposition (abdominal fatty tissue biopsy and/or a positive caridiac 99mTc-diphosphono-1,2-propanodicarboxylic acid (99mTcDPD) scintigraphy if cardiac deposits are assumed). Amyloid confirmation with Congo red staining and immunohistochemical typing were performed according to clinical routine practice at the tertiary referral centre. Alternative causes of neuropathy and cardiomyopathy were excluded through standard clinical evaluation.

Asymptomatic carriers were identified through structured surveillance programs and had no clinical evidence of ATTRv amyloidosis at the time of sampling based on routine neurological and cardiological follow-up assessments.

The genotyping for the control group to confirm the absence of TTR variant p.Val50Met was performed in our own lab by PCR using QuantStudio 6 Flex System thermocycler (Thermo Fisher Scientific) and custom TaqMan SNP genotyping assays (ThermoFischer Scientific).

**Table 1:** Study population characteristics

Group/Genotype	Sex	*N*	Mean Age (Years)	Standard Deviation Age (+/-Years)	Minimum Age (Years)	Maximum Age (Years)
Symptomatic TTR p.V50M carriers	Female	51	63.29	12.45	24.61	86.22
	Male	114	61.16	13.62	29.5	82.9
Asymptomatic TTR p.V50M carriers	Female	34	60.4	9.68	42.47	84.61
	Male	35	60.08	12.37	33.21	92.37
Non-carrier controls	Female	41	50.01	18.24	21.75	79.82
	Male	29	59.07	16.35	21.29	87.78
Total	Female	126	58.19	15.07	21.75	86.22
	Male	178	60.6	13.81	21.29	92.37
	All	304				

Description of the study population’s size, age and sex distribution, divided into three groups symptomatic and asymptomatic TTR p.V50M carriers and non-carrier controls

### Quantification of relative mtDNA copy number

Relative quantification of mtDNA copy number was done by Real-Time quantitative PCR (qPCR) using QuantStudio 6 Flex System thermocycler (Thermo Fisher Scientific) and subsequently analysed using QuantStudio Real-Time PCR Software (Thermo Fisher Scientific). The resulting qPCR replicate data was given as Cq values (quantification cycle), describing how many DNA replicate cycles the qPCR reaction goes through to reach a set threshold. qPCR was performed using TaqMan gene expression assays containing TaqMan hydrolysis probes (final respective probe concentrations of 1X) and TaqMan multiplex master mix (final concentration of 1X) (Thermo Fischer Scientific). The two single copy genes used were the nuclear reference gene *β2M* (Hs06637353_s1 with VIC hydrolysis probe) and the mitochondrial gene of intererest *MT-ND2* (Hs02596874_g1 with FAM hydrolysis probe) (Thermo Fischer Scientific). The amplification efficiency of both the mitochondrial and nuclear primer was controlled through a dilution series comparison of respective R^2^ values, confirming they were equal enough to perform well in a multiplex reaction. Final DNA concentration used in the assay was 0.5 ng/µl. Resulting reaction volume was 10 µl. 96 well plates (Microamp) were used for the qPCR reactions. All qPCR reactions were performed in triplicate and each plate analysis included inter-assay controls to minimise technical variability between runs. A coefficient of variation (CV) of < 10% between the inter-assay controls was considered an acceptable variation between the plate batches.

The relative mtDNA copy number was determined by first calculating the fold change using the established 2^(-ΔΔCq)^ method [30]. The reference *β2M*-gene mean replicate data was used as the reference value to which the target value was normalised, the target value being the mitochondrial *MT-ND2*-gene mean replicate data. Further, the mean ΔCq of control group was used as a calibrator, of which each calculated sample fold change value was divided by. Finally, to achieve a relative mtDNA copy number of TTR. pV50M carriers relative to a healthy non-carrier population, each 2^(-ΔΔCq)^ sample value was divided by the mean 2^(-ΔΔCq)^ of the control group [31]. Thus, the 2^(-ΔΔCq)^ method resulted in a value describing the relative mtDNA copy number of a TTR p.V50M carrier group, symptomatic and asymptomatic, respectively, compared to the control group, where a relative mtDNA copy number of 1 would mean no difference compared to the non-carrier control mean.

### Statistical analysis

Statistical analysis of the relative mtDNA copy number, given as 2^(-ΔΔCq)^ values (fold change), was performed using SPSS Statistics software version 29.0.1.0 (171) (IBM). 05. To assess differences between groups while adjusting first for age, then for age and sex, an ANCOVA was conducted, with mtDNA copy number as the dependent variable and age and sex included as covariates. Pairwise post-hoc tests were corrected for multiple testing using sidak correction. Statistical significance was considered at *p* < 0.05. The three groups compared were symptomatic and asymptomatic TTR p.V50M carriers, and non-carrier controls, and they were analysed first unstratified, then stratified by sex, and stratified by Swedish early- and late-onset age (< 50 years and ≥ 50 years).

## Results

QPCR was performed on the study population’s DNA samples (*n* = 304), which were stratified into three groups: symptomatic TTR p.V50M carriers (*n* = 165), asymptomatic TTR p.V50M carriers (*n* = 69), and carrier-negative controls (*n* = 70). The groups were first compared with mixed genders, not stratifying for sex, to explore mtDNA copy number differences at the general Swedish ATTRV50M population level. Overall comparison effect sizes, reported as partial eta squared (η_p_^2^), as well as mean differences between groups and corresponding 95% confidence intervals for all comparisons can be found in Table 3.

**Fig. 1:**
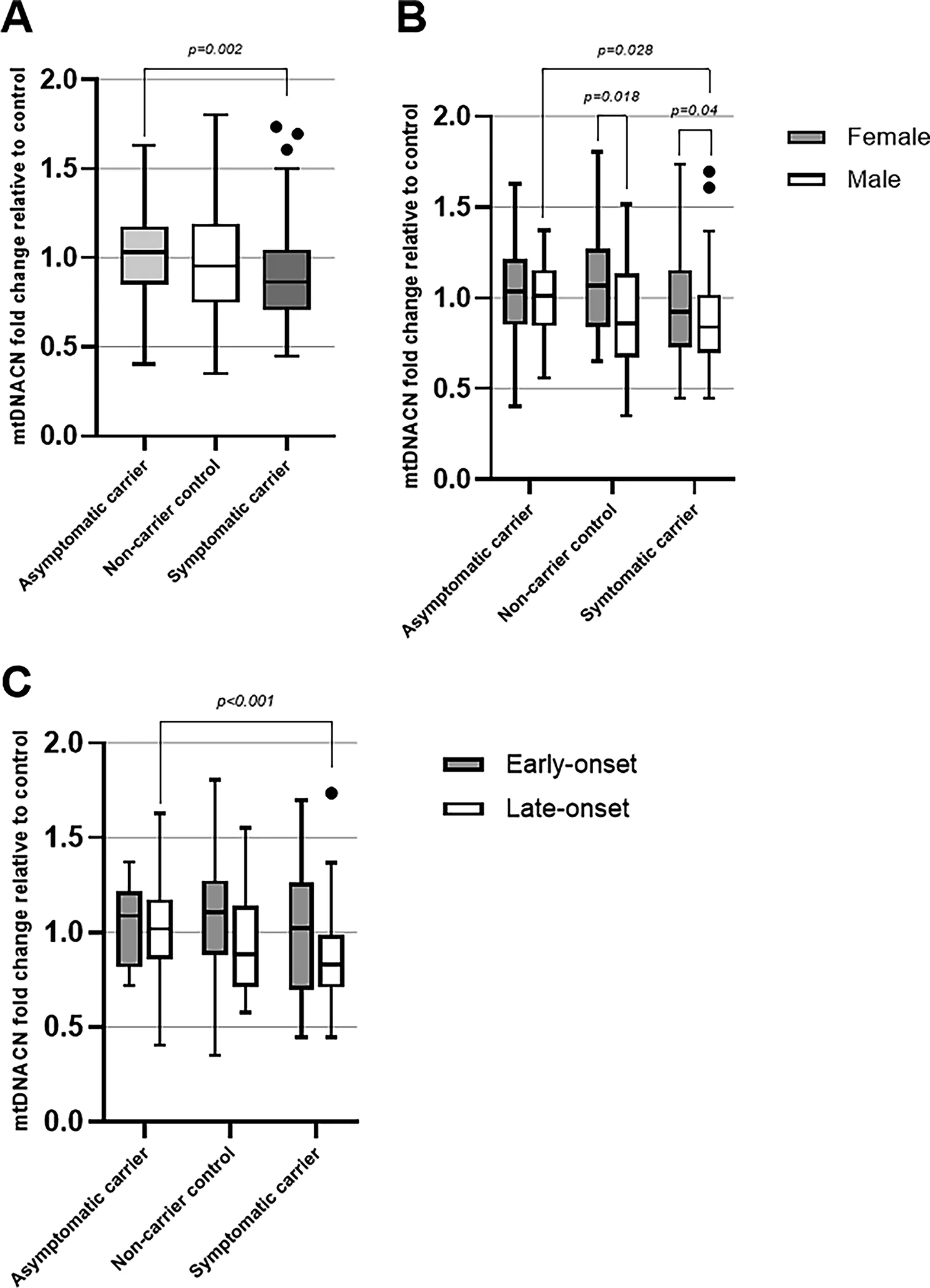
Relative mitochondrial DNA copy number levels adjusted for age. Box plots visualizing the relative mitochondrial DNA copy number (expressed as 2^(−ΔΔCq)^_Group_/2^(−ΔΔCq)^_Control_) between the three different groups: symptomatic ATTRV50M carriers, asymptomatic ATTRV50M carriers, and healthy ATTRV50M-negative controls (**A**), stratified by sex (Female, Male) (**B**), and stratified by Swedish early- and late-onset age (< 50 year-olds and ≥ 50 year-olds) (**C**). Significant differences (*p* < 0.05) were analysed using ANCOVA with sidak correction, adjusted for age. The box shows the values falling within the 25th to 75th percentile and the vertical line indicates the median. The whiskers (Tukey) indicate values falling beyond the 25th percentile + 1.5*IQR (interquartile range), and beyond the 75th percentile + 1.5*IQR. Outliers are indicatied as individual points

The age adjusted ANCOVA with sidak correction showed a significant difference was found between the ATTRV50M symptomatic and asymptomatic groups (*p* = 0.002), where symptomatic carriers had a lower mtDNA copy number (Fig. 1A, Table 3A). When stratified by sex, a significantly lower mtDNA copy number in symptomatic compared to asymptomatic carriers was observed only among males (*p* = 0.028) (Fig. 1B, Table 3B). When analysing the mtDNA copy number differences between males and females in each of the three groups symptomatic carriers, asymptomatic carriers, and non-carrier controls, only in the non-carrier and symptomatic carrier group did females show a significantly higher copy number than males (*p* = 0.018 and *p* = 0.04, respectively) (Fig. 1B, Table 3C).

**Fig. 2:**
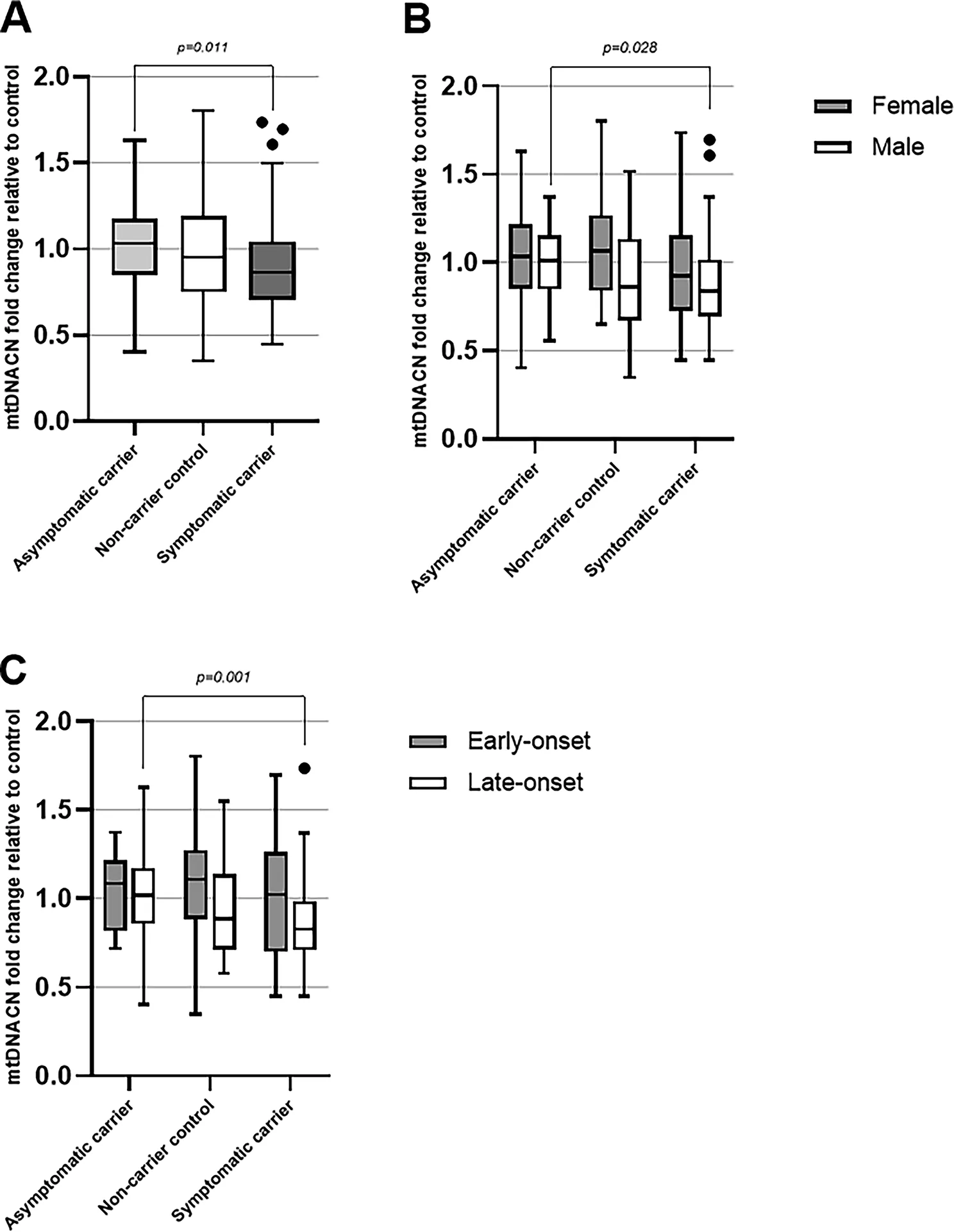
Relative mitochondrial DNA copy number levels adjusted for age and sex. Box plots visualizing the relative mitochondrial DNA copy number (expressed as 2^(−ΔΔCq)^_Group_/2(^−ΔΔCq)^_Control_) between the three different groups: symptomatic ATTRV50M carriers, asymptomatic ATTRV50M carriers, and healthy ATTRV50M-negative controls (**A**), stratified by sex (Female, Male) (**B**), and stratified by Swedish early- and late-onset age (< 50 year-olds and ≥ 50 year-olds) (**C**). Significant differences (*p* < 0.05) were analysed using ANCOVA with sidak correction, adjusted for age and sex. The box shows the values falling within the 25th to 75th percentile and the vertical line indicates the median. The whiskers (Tukey) indicate values falling beyond the 25th percentile + 1.5*IQR (interquartile range), and beyond the 75th percentile + 1.5*IQR. Outliers are indicatied as individual points

The ANCOVA analyses were repeated, this time adjusting for both age and sex (Fig. 2B) as a robustness check to see whether the observed sex differences are still significant after adjusting for sex as well as age. Males continued to show a significant difference (*p* = 0.028) between the symptomatic and asymptomatic carrier groups, similarly to the unstratified age adjusted analysis (Fig. 2B, Table 3G). A following unstratified age and sex adjusted ANCOVA (Fig. 2A, Table 3F) showed the almost the same significant difference the symptomatic and asymptomatic carrier groups with a slightly lower p-value (*p* = 0.011). This could suggest that symptomatic TTR p.V50M carriers have significantly lower mtDNA copy number levels than symptomatic carriers, especially in males, where this difference is associated with symptomatic or asymptomatic status and not only biological sex differences in mtDNA levels.

Finally, to assess differences between early- and late-onset aged carriers, which is under, respectively above, 50 years of age in Sweden, symptomatic carriers were stratified into two groups, whether they were < 50 years old or ≥ 50 years old when symptoms started. Asymptomatic carriers and non-carrier controls were stratified into two age groups (< 50 year-olds and ≥ 50 year-olds). Again, symptomatic carriers were revealed to have a lower copy number than asymptomatic carriers (*p* < 0.001), but only for the late-onset individuals (Fig. 1C, Table 3D). It is important to note that the age of onset and the age of sampling was not the same in all symptomatic individuals because age of onset could sometimes be established years before official diagnosis and blood sampling (Table 2). An age and sex adjusted ANCOVA (Fig. 2C, Table 3H) showed similarly that late-onset symptomatic carriers had a lower copy number than late-onset asymptomatic carriers (*p* = 0.001), suggesting that age and sex are likely not influencing the indicated mtDNA copy number differences.

**Table 2:** Study Population Characteristics by Age of Onset

Group/Genotype	Early/Late Age of Onset (< 50/≥50 years)	*N*	Mean Age (Years)	Standard Deviation Age (+/-Years)	Minimum Age (Years)			Maximum Age (Years)
Symptomatic TTR p.V50M carriers	< 50	42	43,79	9,81	25			69
	≥ 50	123	68,05	7,26	53			86
Asymptomatic TTR p.V50M carriers	< 50	10	43,00	5,54	33			49
	≥ 50	59	63,19	8,89	50			92
Non-carrier Control	< 50	27	35,07	10,58	21			49
	≥ 50	43	65,49	9,81	51			88
	All	304						

Description of the study population’s size, age and sex distribution, divided into three groups symptomatic and asymptomatic TTR p.V50M carriers and non-carrier controls, and stratified by early- and late-onset age (< 50 year-olds and ≥ 50 year-olds)

**Table 3:** ANCOVA test effect sizes, mean differences between groups and corresponding 95% confidence intervals

ANCOVA test	Group stratification	Group Comparison (a vs. b)	Mean MtDNA fold change Difference (a-b)	Significance (*p*-value)	95% CI	Overall test F-value, *p*-value, and effect size (partial eta squared, η_*p*_^2^)
A) Age adjusted		Asymptomatic vs. Symptomatic	0.127	0.002*	[0.037,0.217]	F(2,300) = 6.235; *p* = 0.008; η_p_^2^ = 0.040
		Asymptomatic vs. Non-carrier	0.053	0.556	[-0.055,0.161]	
		Non-carrier vs. Symptomatic	0.074	0.152	[-0.018,0.55]	
B) Age adjusted	Female	Asymptomatic vs. Symptomatic	0.101	0.293	[-0.050,0.252]	F(2,122) = 1.475; *p* = 0.233; η_p_^2^ = 0.024
		Asymptomatic vs. Non-carrier	0.023	0.98	[-0.141,0.188]	
		Non-carrier vs. Symptomatic	0.077	0.538	[-0.77,0.232]	
	Male	Asymptomatic vs. Symptomatic	0.122	0.028*	[0.010,0.234]	F(2,174) = 3.517; *p* = 0.032; η_p_^2^ = 0.039
		Asymptomatic vs. Non-carrier	0.112	0.184	[-0.034,0.257]	
		Non-carrier vs. Symptomatic	0.01	0.996	[-0.111,0.131]	
C) Age adjusted	Asymptomatic	Female vs. Male	0.067	0.273	[-0.054,0.189]	F(1,66) = 1.223; *p* = 0.273; η_p_^2^ = 0.018
	Symptomatic	Female vs. Male	0.088	0.040*	[0.004,0.171]	F(1,162) = 4.285; *p* = 0.040; η_p_^2^ = 0.026
	Non-carrier	Female vs. Male	0.171	0.018*	[0.030,0.312]	F(1,67) = 5.894; *p* = 0.018; η_p_^2^ = 0.081
D) Age adjusted	Early onset	Asymptomatic vs. Symptomatic	0.04	0.974	[-0.216,0.296]	F(2,75) = 0.104; *p* = 0.902; η_p_^2^ = 0.003
		Asymptomatic vs. Non-carrier	0.013	0.999	[-0.265,0.290]	
		Non-carrier vs. Symptomatic	0.027	0.981	[-0.167,0.221]	
	Late onset	Asymptomatic vs. Symptomatic	0.168	< 0.001*	[0.071,0.264]	F(2,221) = 8.977; p = < 0.001; η_p_^2^ = 0.075
		Asymptomatic vs. Non-carrier	0.082	0.274	[-0.038,0.201]	
		Non-carrier vs. Symptomatic	0.086	0.145	[-0.019,0.192]	
E) Age adjusted	Asymptomatic	Early onset vs. Late onset	-0.121	0.291	[-0.346,0.105]	F(1,66) = 1,135; *p* = 0.291; η_p_^2^ = 0.017
	Symptomatic	Early onset vs. Late onset	0.123	0.103	[-0.025,0.271]	F(1,162) = 2,693; *p* = 0.103; η_p_^2^ = 0.016
	Non-carrier	Early onset vs. Late onset	0.118	3.62	[-0.138,0.374]	F(1,67) = 0.844; *p* = 0.362; η_p_^2^ = 0.012
F) Age and sex adjusted		Asymptomatic vs. Symptomatic	0.109	0.011*	[0.020,0.199]	F(2,299) = 4.329; *p* = 0.014; η_p_^2^ = 0.028
		Asymptomatic vs. Non-carrier	0.062	0.417	[-0.045,0.168]	
		Non-carrier vs. Symptomatic	0.048	0.521	[-0.45,0.140]	
G) Age and sex adjusted	Female	Asymptomatic vs. Symptomatic	0.101	0.293	[-0.050,0.252]	F(2,122) = 1.475; *p* = 0.233; η_p_^2^ = 0.024
		Asymptomatic vs. Non-carrier	0.023	0.98	[-0.141,0.188]	
		Non-carrier vs. Symptomatic	0.077	0.538	[-0.077,0.232]	
	Male	Asymptomatic vs. Symptomatic	0.122	0.028*	[0.010,0.234]	F(2,174) = 3.517; *p* = 0.032; η_p_^2^ = 0.39
		Asymptomatic vs. Non-carrier	0.112	0.184	[-0.034,0.257]	
		Non-carrier vs. Symptomatic	0.01	0.996	[-0.111,0.131]	
H) Age and sex adjusted	Early onset	Asymptomatic vs. Symptomatic	0.18	0.998	[-0.240,0.275]	F(2,74) = 0.072; *p* = 0.931; η_p_^2^ = 0.002
		Asymptomatic vs. Non-carrier	0.042	0.978	[-0.239,0.323]	
		Non-carrier vs. Symptomatic	-0.024	0.99	[-0.238,0.190]	
	Late onset	Asymptomatic vs. Symptomatic	0.15	< 0.001*	[0.054,0.246]	F(2,220) = 7.163; p = < 0.001; η_p_^2^ = 0.061
		Asymptomatic vs. Non-carrier	0.081	0.261	[-0.36,0.198]	
		Non-carrier vs. Symptomatic	0.069	0.308	[-0.036,0.173]	
I) Age and sex adjusted	Asymptomatic	Early onset vs. Late onset	-0.124	0.278	[-0.349,0.102]	F(1,65) = 1,1.197; *p* = 0.278; η_p_^2^ = 0.018
	Symptomatic	Early onset vs. Late onset	0.115	0.123	[-0.032,0.262]	F(1,161) = 2,402; *p* = 0.123; η_p_^2^ = 0.015
	Non-carrier	Early onset vs. Late onset	0.095	0.451	[-0.154,0.343]	F(1,66) = 0.576; *p* = 0.451; η_p_^2^ = 0.009

This table presents the all the group comparisons’ mean differences in relative mtDNA copy number, the corresponding overall test p=values, corresponding 95% confidence intervals (CI), as well as overall ANCOVA tests’ (A-E age adjusted, F-I age and sex adjusted) effect sizes, reported as partial eta squared (η_p_^2^). Significant p-values (*p* < 0.05) are marked with an asterisk (*)

## Discussion

In this study, by performing an age adjusted ANCOVA we demonstrate that symptomatic Swedish TTR p.V50M carriers exhibit a significantly lower mitochondrial DNA (mtDNA) copy number in peripheral blood cells compared to asymptomatic carriers. Our results support a potential association between mitochondrial dysfunction and symptomatic disease, rather than carrier status per se. A Portuguese ATTRV50M amyloidosis cohort previously demonstrated increased mtDNA copy number particularly among early-onset carriers. In contrast, our Swedish cohort demonstrated lower mtDNA copy number in symptomatic carriers, particularly among later-onset individuals. However, similarly to the study on the Portuguese cohort which showed no significant differences in mtDNA copy number when comparing late- to early-onset individuals despite a trend of higher mean mtDNA copy number in the early-onset group, our Swedish cohort showed no such differences either. Although the observed mtDNA patterns differed between populations, both studies support a potential association between mtDNA copy number and age-at-onset variability in ATTRV50M amyloidosis [27].

The present findings may be interpreted in the context of previous observations suggesting a role of mitochondrial biology in ATTR amyloidosis. Earlier studies from our group demonstrated that mitochondrial haplotypes are associated with age of onset and disease phenotype in Swedish and French carriers of TTR p.Val50Met, indicating that inherited mitochondrial factors may influence disease expression [32]. Furthermore, mitochondrial genetic variation has been proposed as potential contributor to the parent-of-origin effects observed in ATTRv amyloidosis, where maternal transmission is associated with increased penetrance and earlier disease onset [33]. In addition, previous studies from Swedish ATTRv families have demonstrated marked heterogeneity in disease penetrance and significantly higher penetrance following maternal transmission of the pathogenic variant [34]. Together, these findings support the hypothesis that mitochondrial mechanisms may contribute to the variability of disease expression in ATTRv amyloidosis, although the underlying biological pathways remain incompletely understood.

MtDNA copy number is tissue specific, and intracellular mtDNA levels may decrease under conditions of oxidative stress, potentially due to cellular export or degradation [35, 36]. While this has been described in other disease contexts [37–40], the relevance of extracellular mtDNA in ATTRV50M remains to be determined. In addition, mtDNA copy number measured from buffy coat-derived DNA may be influenced by leukocyte subtype composition and other pre-analytical factors. Complete blood count variables and leukocyte subtype distributions were unfortunately not available in this cohort, and residual confounding related to cellular composition therefore cannot be excluded.

MtDNA copy number represents only one aspect of mitochondrial and redox homeostasis, and future studies incorporating complementary oxidative stress markers will be necessary to clarify underlying mechanisms [41–44].

Another perspective relates to the strictly maternal inheritance of mtDNA. Prior studies have suggested higher penetrance when the variant TTR genotype is inherited from the mother, particularly in male offspring. Although our study was not designed to directly test inheritance patterns, the combination of maternal inheritance and observed sex differences in mtDNA copy number points to an area for future research. Systematic studies on inheritance effects may help clarify whether mitochondrial factors contribute to variability in disease penetrance and onset.

Oxidative stress and inflammation are closely interconnected biological processes. Previous studies have reported elevated inflammatory markers in ATTRv amyloidosis, including in presymptomatic carriers [45], suggesting that inflammatory pathways may contribute to the altered redox environment observed in the disease. However, inflammatory markers were not assessed in the present study and the potential relationship between inflammation and mtDNA copy number therefore warrants further investigation.

When stratifying our study population by sex, only male participants exhibited the same copy number differences as the unstratified population. When comparing mean mtDNA number between sexes to evaluate potential sex associated differences within carrier groups, female participants exhibited a trend of higher mean mtDNA copy numbers compared to males across all groups, though a statistically significant difference was only observed in the the symptomatic carrier group and the non-carrier group. An additional age and sex adjusted ANCOVA showed that males continued to exhibit the same difference as the unstrantified analysis, while the within-group differences between the sexes disappeared. This could indicate that the observed mtDNA copy number difference between symptomatic and asymptomatic carriers is more apparent among males in this cohort. However, the study was not powered to formally evaluate sex interactions and these findings should therefore be interpreted cautiously. Although sex-stratified analyses reduce statistical power, these findings may indicate sex-related differences in mtDNA copy number that warrant further investigation. This could be of potential importance especially when considering sex distribution in the interpretation of population-level analyses in ATTRV50M [46, 47]. However, the study was not powered to formally evaluate sex interactions and these findings should therefore be interpreted cautiously.

It is also important to note that age is correlated with lower mtDNA copy number in healthy individuals. Though consistent world-wide age reference values have not been developed yet, findings show that mtDNA levels can start to decline after age of 50 [48, 49]. Our study groups mean ages were significantly different when comparing symptomatic carriers with the carrier negative control group (*p* = 0.001) (results not shown). The mean age of symptomatic carriers was 61.3 while the mean age of control group was 53.8. Age related mtDNA copy number differences were therefore accounted for by performing age adjusted statistical analyses.

Taken together, our findings indicate that reduced mtDNA copy number could be associated with symptomatic ATTRV50M amyloidosis in the Swedish population. While causality cannot be inferred, the observed differences suggest mitochondrial involvement in disease expression and emphasize the need for longitudinal and mechanistic studies to determine whether mtDNA alterations precede or follow symptom onset.

## Conclusions

This study showed a significantly lower mtDNA copy number in symptomatic carriers of ATTRV50M when compared to a non-carrier control group, as well as when compared to the asymptomatic ATTRV50M group. A similar significant difference could not be seen when comparing the asymptomatic ATTRV50M group to the control group. Though this study cannot offer a conclusive mechanism explaining these findings, it supports a potential association between altered mitochondrial biology and symptomatic disease status in ATTRv amyloidosis.

With this study we are one step closer to answering this question while increasing our understanding of the disease. These findings may contribute to future studies aimed at improving early diagnosis and optimizing treatment strategies in ATTRv amyloidosis. Looking ahead, our findings also raise several questions that warrant further investigation into the role of mitochondria and inheritance patterns in ATTRV50M.

### Future perspectives

A natural next step would be to perform longitudinal studies that combine mtDNA copy number measurements with markers of oxidative stress and redox balance, as well as detailed analyses of maternal versus paternal inheritance patterns. Such studies could clarify whether mitochondrial function and inheritance jointly influence disease penetrance and onset, and may ultimately strengthen the role of mtDNA as a predictive biomarker in ATTRV50M.

External factors such as diet and environmental exposures may influence oxidative stress and mitochondrial function, and could therefore contribute to inter-individual variability in mtDNA copy number [50–53]. Future studies integrating mtDNA analyses with metabolic and environmental biomarkers may help clarify whether such factors modify disease expression in ATTRV50M. Similarly, redox-homeostatis disruptive factors such as chronic inflammation in addition to carrier and phenotype status could be important additional biomarkers to analyse.

## Data Availability

The data from this study may be obtained from the corresponding author upon reasonable request.

## Abbreviations

AD: Alzheimer’s disease
ALS: Amyotrophic lateral sclerosis
ATP: Adenosine triphosphate
ATTR: Transthyretin amyloidosis
ATTRv: Hereditary (or variant) transthyretin amyloidosis
Cq: Quantification cycle
DNA: Deoxyribonucleic acid
MS: Multiple sclerosis
mtDNA: Mitochondrial deoxyribonucleic acid
PCR: Polymerase chain reaction
PD: Parkinson’s disease
qPCR: Real-time quantitative polymerase chain reaction
RNA: Ribonucleic acid
RNS: Reactive nitrogen species
ROS: Reactive oxygen species
TTR: Transthyretin
